# The association of integration patterns of human papilloma virus and single nucleotide polymorphisms on immune- or DNA repair-related genes in cervical cancer patients

**DOI:** 10.1038/s41598-019-49523-0

**Published:** 2019-09-11

**Authors:** Jungnam Joo, Yosuke Omae, Yuki Hitomi, Boram Park, Hye-Jin Shin, Kyong-Ah Yoon, Hiromi Sawai, Makoto Tsuiji, Tomonori Hayashi, Sun-Young Kong, Katsushi Tokunaga, Joo-Young Kim

**Affiliations:** 1 Biometrics Research Branch, National Cancer Center, Goyang, Korea; 2 Department of Human Genetics, Graduate School of Medicine, The University of Tokyo, Tokyo, Japan; 3 Genome Medical Science Project (Toyama), National Center for Global Health and Medicine (NCGM), Tokyo, Japan; 4 Department of Microbiology, Hoshi University School of Pharmacy and Pharmaceutical Sciences, Tokyo, Japan; 5 Particle Therapy Research Branch, National Cancer Center, Goyang, Korea; 6 College of Veterinary Medicine, Konkuk University, Seoul, Korea; 7 Center for Breast Cancer, National Cancer Center, Goyang, Korea; 8 Department of Radiobiology and Molecular Epidemiology, Radiation Effects Research Foundation, Hiroshima, Japan; 9 Translational Research Branch, National Cancer Center, Goyang, Korea

**Keywords:** Tumour virus infections, Genetic association study, Viral infection

## Abstract

The present study investigated the association between single nucleotide polymorphisms (SNPs) in immune- or DNA repair-related genes and the integration pattern of human papillomavirus (HPV), a promising prognostic marker in cervical cancer. The HPV integration patterns of cervical cancer patients were determined by polymerase chain reaction and *in situ* hybridization, and categorized as episomal (group A), single-copy or multi-copy tandem repetition integrated (group B), and undetectable HPV types (group C). After sample and SNP quality control, 166,505 SNPs in 161 samples (38, 111, and 12 patients in groups A, B, and C, respectively) were examined. None of the SNPs reached genome-wide significance, and several candidate SNPs for future study were selected, including rs10999435 on chromosome 10q22, rs1322054 on chromosome 9q32-33, and rs10902171 on chromosome 11p15. Luciferase assay identified rs1322054 as the primary functional variant to regulate gene expression in immune cell. Further studies are needed to determine the genetic background of different integration patterns of HPV in cervical cancer patients.

## Introduction

Current standard treatment for patients with locally advanced cervical cancer is radiotherapy with concurrent chemotherapy. However, the response to radiotherapy varies widely among patients, underscoring the need to identify biomarkers to predict patients’ treatment outcome. Recently, the integration pattern of human papillomavirus (HPV) has been investigated as a promising biomarker for cervical cancer patients^1^.

Integration of HPV DNA into the host genome is associated with cervical carcinogenesis. However, in our previous study, HPV DNA is present in episomal form in approximately 20% of patients with locally advanced cervical cancer^2^. The survival rate is higher in patients with tumors containing abundant episomal HPV DNA than in those with completely integrated HPV DNA or no HPV DNA^2^. We hypothesized that impairment of the DNA damage response and immune response, which affect the response to radiotherapy, may be associated with the HPV integration pattern. The bases of this hypothesis are as follows. First, the presence of high-risk HPV for a long period of time is dependent on its resistance to immune clearance mechanisms in the body, and episomal HPV DNA may be differently targeted by the immune surveillance system. High-risk HPV develops several mechanisms to avoid host immune response and persists as episomes in the HPV-infected cells in transformation zone of the cervix before progression to cervical cancer. One of the strategies to avoid detection is to prevent pro-inflammatory signal by blocking recruitment of antigen-presenting cells and the release of cytokines that mediate the immune response. In W12 keratinocyte model where HPV16 is naturally infected into normal keratinocyte and sustain as episomal status, it was shown that HPV16- integrated cells emerged at the same time episomes are cleared, and complete deregulation of viral oncogenes did not occur without complete reduction of E2, which is expressed by viral episomes. Gene expression comparison between the W12 cells with episomal HPV and integrated HPV by microarray analysis showed that disappearance of episome was associated with rapid increase in type I interferon pathway with rapid change in genes related to antiviral response^3^. In the same W12 model, it was also shown that episomal clearance caused by interferon-beta resulted in de-repression of viral integrants and hastened changes of HPV physical status from episomes to integrant^4^. The persistence of episomal HPV DNA in transformed cells provides high amounts of viral antigen through active replication, particularly E2. These cells are preferentially targeted by the immune system^5^ compared with cells with no or lower rates of viral replication. The viral antigen E2 is a major target of T cells during HPV infection^6^. Second, some individuals may be more vulnerable to HPV integration, which requires homologous recombination (HR) between HPV and the host genome^7^. These individuals may be similarly vulnerable to radiation-induced cell killing, because both HPV integration and repair of radiation-induced DNA breakage involve homologous recombination repair. HPV genome integration is frequently observed in important DNA repair genes such as Rad51B^8^, supporting the relationship between DNA damage repair and HPV integration. In a previous study, we showed that genetic variants of excision base repair proficiency genes are strongly associated with cervical cancer compared with the healthy control population. This finding also suggests that an individual host’s base excision repair or nucleotide excision repair ability may facilitate HPV viral persistence and cervical carcinogenesis^9^. Based on these, we hypothesized that genomic susceptibility to DNA damage may explain the association between differential HPV integration and radiotherapy outcome in cervical cancer patients.

Here, we investigated the potential relationship between genomic variants of DNA damage repair- and immune-related genes and different HPV integration patterns using a customized single nucleotide polymorphism (SNP) array system.

## Materials and Methods

### Study population

A comprehensive association study of immune- and DNA repair-related genes was performed in 192 advanced cervical cancer patients treated by radiotherapy ± chemotherapy between July 2003 and August 2010 at the National Cancer Center, Korea. In a recent study^1^, we observed that the combination of *in situ* hybridization and PCR is effective for discriminating the different integration patterns of HPV DNA.

### Custom-made chip: number of genes and SNPs

A PubMed search identified 1,284 candidate immune-related or DNA repair-related genes, which were used to generate a custom immune chip. Then, 981,980 single nucleotide variants located within 50 kpb upstream and downstream of the targeted genes were identified, and 561,049 SNPs validated by Affymetrix were loaded on the custom-made array.

### Genotyping and sample quality control

DNA was extracted from whole peripheral blood using a QIAamp DNA Blood Mini Kit (Qiagen, Valencia, CA) according to the manufacturer’s instructions. For the genetic association study, 192 samples were genotyped using the custom immuno-array described above according to the manufacturer’s instructions for the Affymetrix Axiom Array platform. All samples passed a sample quality control (QC) metric with a Dish QC > 0.82, and genotype calling was performed using Genotyping Console v4.2 software. All samples passed an overall call rate threshold >97%, and the average overall call rate was 99.71% (99.19–99.87%). No duplicates or first-degree relatives were detected in identity-by-descent testing and principal component analysis using HapMap data as controls showed that all Korean cervical cancer samples formed a single cluster together with the HapMap Asian population samples (Japanese in Tokyo [JPT] and Han Chinese in Beijing [CHB]) (Fig. S1).

### SNP quality control

Several SNP QC steps were performed before applying the association test. Monomorphism (n = 313,993) and loss of heterozygosity (n = 270) were first assessed, and SNPs with a call rate ≤ 95% (n = 6,596) and a minor allele frequency ≤ 5% (n = 72,427) were excluded. Finally, SNPs that deviated from Hardy–Weinberg Equilibrium (p ≤ 0.001) (n = 1,258) were further filtered out, resulting in the inclusion of 166,505 SNPs on autosomal chromosomes or X chromosome in the association test. All cluster plots for SNPs with *p*-values < 5E-04 from the association test were checked by visual inspection, and SNPs with ambiguous genotype calls were excluded. Candidate regions for further analysis were selected using a *p*-value threshold of 5E-04 (Table S1).

### HPV integration pattern

The integration pattern of 30 patients could not be determined because PCR results were not available for these patients. Moreover, duplicated result was found for one patient. After excluding these patients, 161 patients were included for further analysis. The integration pattern of patients’ HPV was classified as episomal (group A), single-copy or multi-copy tandem repetition integrated (group B), and undetectable HPV (group C)^1^. According to this criterion, 38 (23.6%), 111 (68.9%), and 12 (7.5%) of 161 patients were classified into groups A, B, and C, respectively.

### Statistical analysis

The distribution of patient characteristics was summarized as proportions for categorical variables and median (range) for continuous variables. The differences between groups were tested using Pearson’s Chi-squared or Fisher’s exact test for categorical variables and Wilcoxon rank-sum or Kruskal–Wallis test for continuous variables as appropriate. Differences in the genotype distribution of SNPs between HPV integration patterns were tested using additive and dominant genetic models, and the smallest p-value in the two tests was used to order the significance of the association of SNPs^10^. Tumor size, which was the only significant variable at p < 0.05 in all logistic regression models, was adjusted in the logistic regression models before evaluating the association of SNPs with outcome.

### *In silico* analysis of candidate variants

Potential functional variants for transcription regulation were evaluated using the RegulomeDB database (http://www.regulomedb.org/index)^11^. The expression of candidate genes was searched on the Human Protein Atlas database (http://www.proteinatlas.org)^12^. The correlation between the SNP genotype and the expression of surrounding genes was examined using available data from the GTEx portal (http://gtex-portal.org/home/)^13^, DICE (https://dice-database.org/)^14^, blood eQTL browser (https://molgenis58.target.rug.nl/bloodeqtlbrowser/)^15^, and iMETHYL (http://imethyl.iwate-megabank.org/)^16^. The possible impact of nonsynonymous variants on the structure and function of proteins was evaluated using the PolyPhen-2 tool (http://genetics.bwh.harvard.edu/pph2/)^17^.

### Luciferase reporter assay

Specific PCR primers (Table S2) were used to amplify the sequences including each allele of rs1322054, each allele of rs10999435, and major allele of rs112360405, from human genomic DNA. Amplicons were then subcloned into the pCR-bluntII vector (Thermo-Fisher Scientific, Waltham, MA). The sequences of the constructs were confirmed by sanger sequencing using 3730xl DNA analyzer (Thermo-Fisher Scientific) (Fig. S2). Minor allele of rs112360405 was constructed by PCR-directed mutagenesis using allele-specific primer sets (Table S2), and confirmed by sanger sequencing (Fig. S2). Each DNA fragment including each allele of every SNPs was then subcloned into the luciferase reporter pGL4.23 (luc2/minP) vector (Promega, Madison, Wis), using Sac I and Xho I sites (rs1322054, rs10999435, and forward direction of rs112360405) or Hind III and Xho I sites (reverse direction of rs112360405) (Fig. S3). pGL4.23 constructs of each allele (1ug) and the pGL4.74 (hRluc/TK) vector (100 ng), which was used as an internal control, were transfected into Jurkat cell using Lipofectamine 3000 (Thermo-Fisher Scientific). For the measurement of luciferase activity, the Dual-Luciferase Reporter Assay system (Promega, Madison, WI) was used. Differences in relative luciferase activity were compared between major and minor alleles of each SNP using Student’s t test. P values < 0.05 were regarded as statistically significant. Three independent experiments were performed in each assay.

### Ethics statement

The protocol of the present study was approved by the Ethics Committee of the Graduate School of Medicine, The University of Tokyo, and Internal Review Board of the National Cancer Center, Korea (IRB no. NCCNCS 10-412). All experiments were performed in accordance with the relevant guidelines and regulations. All subjects provided written informed consent.

## Results

### Patient characteristics

Table 1 summarizes the patient characteristics according to HPV integration patterns A, B, and C. The mean (SD) age of the study population was 55.8 (13.8) years. Advanced stage (stage III or higher) and adeno/adenosquamous cell histologic type were detected in 18 (11.2%) and 19 (11.8%) patients, respectively. Poorly differentiated histologic grade was observed in 38 (24.4%) patients, and 90 (55.9%) patients presented with large (≥4 cm) tumor size. Nodal metastasis (positive nodal status) was observed in 92 (57.1%) patients. Most of the patients (131/161, 81.4%) were non-smokers. HPV 16 was the most common type (79%), and HPV types 18 and 58 were observed in 21 (14.7%) and 9 (6.3%) patients, respectively. Tumor size ≥4 cm was observed more frequently in groups C (83.3%) and B (58.6%) than in group A (39.5%). No significant difference in the other variables was observed between groups.

**Table 1:** Distribution of clinical variables according to HPV integration patterns in the GWAS set.

		Total	group A	group B	group C	p-value
		(N = 161)	(N = 38)	(N = 111)	(N = 12)	
**Stage group**						
	~IIB	143	36 (94.74)	96 (86.49)	11 (91.67)	0.449^‡^
	III/IVA	18	2 (5.26)	15 (13.51)	1 (8.33)	
**Histologic grade**						
(miss = 5)	well/moderate	118	30 (81.08)	78 (72.9)	10 (83.33)	0.492^†^
	poor	38	7 (18.92)	29 (27.1)	2 (16.67)	
**Histologic type**						
	SCC	142	34 (89.47)	96 (86.49)	12 (100)	0.510^‡^
	AD/ASC	19	4 (10.53)	15 (13.51)	0 (0.00)	
**Tumor size**						
	<4 cm	71	23 (60.53)	46 (41.44)	2 (16.67)	0.017^†^
	≥4 cm	90	15 (39.47)	65 (58.56)	10 (83.33)	
**Smoking**						
	non-smoker	131	31 (81.58)	90 (81.08)	10 (83.33)	0.981^†^
	present/ex-smoker	30	7 (18.42)	21 (18.92)	2 (16.67)	
**HPV type**						
(miss = 18)	16	113	33 (91.67)	71 (73.96)	9 (81.82)	0.115^‡^
	18	21	1 (2.78)	18 (18.75)	2 (18.18)	
	58	9	2 (5.56)	7 (7.29)	0 (0.00)	
**Nodal status**						
	Node = 0	69	20 (52.63)	45 (40.54)	4 (33.33)	0.338^†^
	1 ≤ node ≤ 5	92	18 (47.37)	66 (59.46)	8 (66.67)	
Age (yr)	mean ± std	161	58.89 ± 12.45	54.25 ± 14.13	60.75 ± 12.44	0.090*
	median (range)	161	59.5 (34–79)	54 (27–83)	58.5 (39–77)	

*p-value, Kruskal–Wallis test.

^†^p-value, Pearson’s Chi-squared test.

^‡^p-value, Fisher’s exact test.

### Genetic association study

To identify SNPs with different genotype distributions between HPV integration patterns, comparisons were made based on the results of logistic regression as follows: (1) A vs. B, (2) A vs. C, (3) B vs. C, and (4) A vs. B + C. In addition, an ordinal regression model was applied using the HPV integration patterns A, B, and C as the ordinal outcome variables. Fig. S4 shows the plots for each association test after adjusting for differences in tumor size. None of the SNPs reached a significance level after Bonferroni correction for the number of tested SNPs (significance threshold α = 3.00E-07 from 0.05/166,505). Therefore, candidate SNPs with a minimum p-value < 5E-04 in the adjusted analysis were selected for the detection of candidate SNPs in subsequent studies (Table S1). Many of the selected SNPs on the same gene were in linkage disequilibrium (LD), and the effective number of SNPs was calculated based on the LD correlation structure^18^.

**Figure 1:**
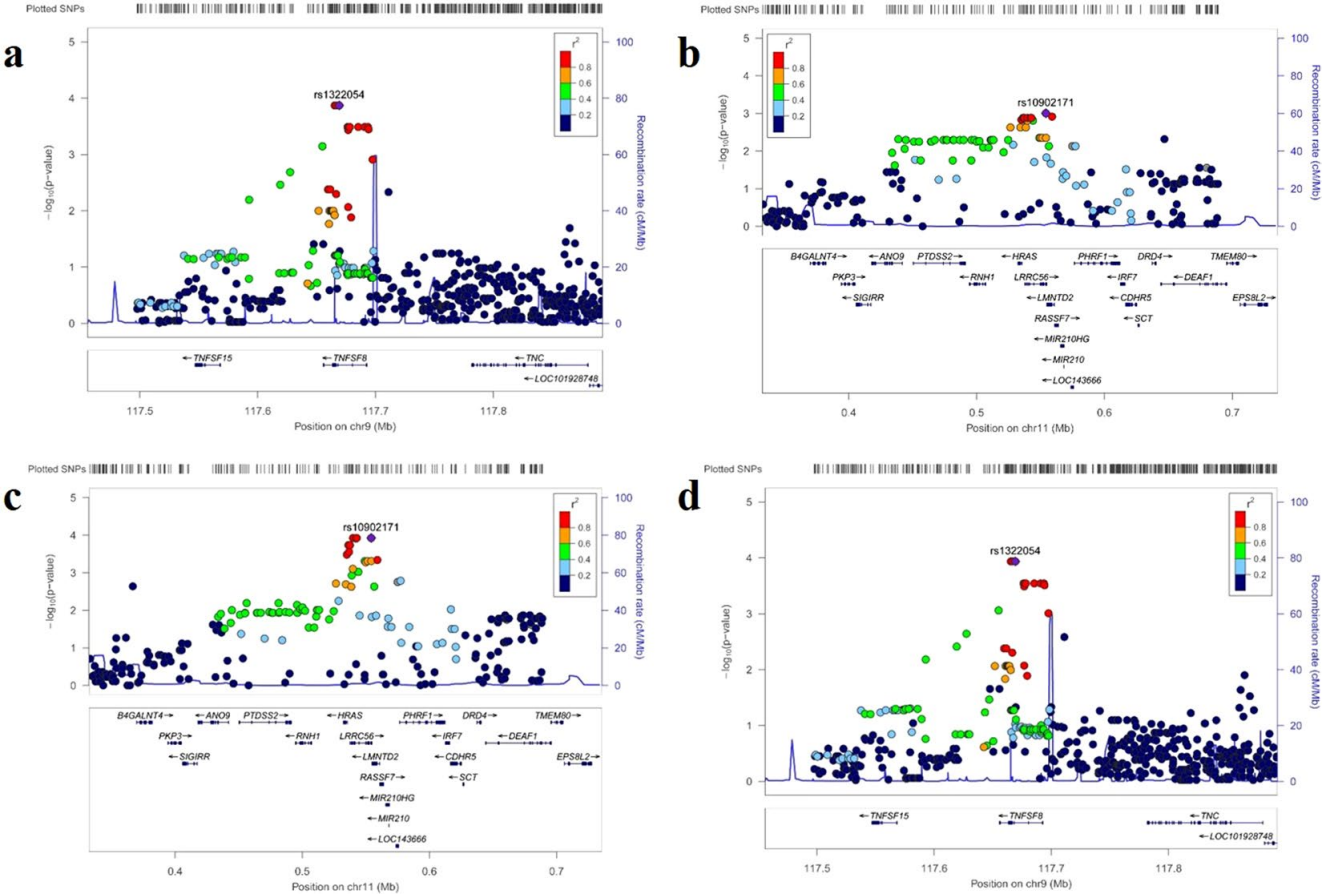
Plot of −log10 (P-value) against the physical location on chromosome 9q32-33 locus (**a,d**), and 11p15 (**b,c**). Each dot represents the −log10 value (p-value) of the respective genotyped SNP. Dots for the most significant SNPs in each locus are marked with a purple diamond, and the color of each dot represents the pairwise r2-value against the top SNPs in 1000 genomes from the Asian population.

Comparison of integration patterns A and B identified rs3181372, rs3181365, and rs1322054 on chromosome 9q32-33 as the SNPs with the smallest p-value at 1.34E-04 (Fig. 1a). These three SNPs were in complete LD, and rs3181372 was located in the 3′-UTR and the other two SNPs were located in the intronic region of the *TNFSF8* gene. In total, 39 SNPs (effective number: 33.6) in 13 loci were significant with a p-value < 5E-04.

Comparison between groups A and C did not identify any significant SNPs with a p-value < 5E-04. This could be attributed to the small sample size of these two groups. Two SNPs, rs10902170 and rs10902171, that were in complete LD on chromosome 11p15 and located near the *HRAS* gene showed an association, with a minimum p-value of 9.89E-04 (Fig. 1b). These SNPs were also located in the *LRRC56* gene, which contains a leucine-rich repeat but whose function is unknown, and resulted in an amino acid substitution arg507-to-gly (R507G) and asp523-to-his (D523H), respectively. The D523H substitution was predicted to be damaging for the LRRC56 protein with a score of 0.983 according to PolyPhen-2.

Comparison of integration patterns B and C identified 13 SNPs (effective number: 4.3) in two loci with a p-value < 5E-04. The minimum p-value was observed for four SNPs in complete LD near the *HRAS* and *LRRC56* genes, with a p-value of 1.19E-04 (Fig. 1c). These SNPs included rs10902170 and rs10902171, which were also identified in the comparison between A and C, and rs74920097 and rs2894649, located at 4.4 kbp and 7.3 kbp 5′ of the *HRAS* gene and the intronic region of the *LRRC56* gene.

Comparison of integration pattern groups A and B + C identified 54 SNPs (effective number: 48.2) in 14 loci with a p-value < 5E-04. The minimum p-value of 1.16E-04 was observed for SNPs rs3181372, rs3181365, and rs1322054, which showed a minimum p-value in the comparison between groups A and B (Fig. 1d).

**Figure 2:**
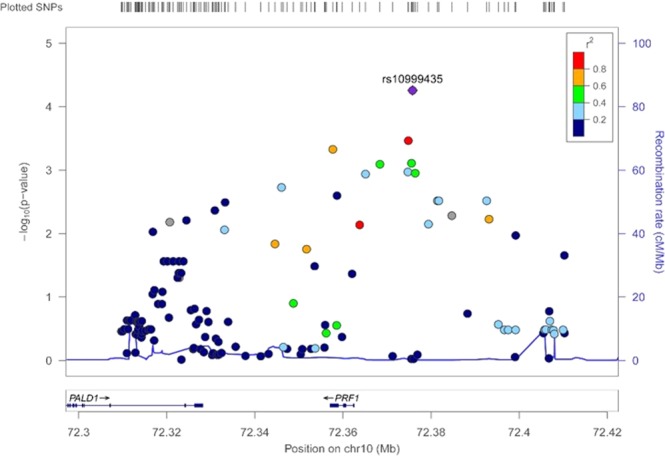
Plot of −log10 (p-value) against the physical location on chromosome 10q22. Each dot represents the −log10 value (p-value) of the respective genotyped SNP. The dot for the most significant SNP is marked with a purple diamond, and the color of each dot represents the pairwise r2-value against the top SNPs in 1000 genomes from the Asian population.

The ordinal comparison of groups A, B, and C identified 84 SNPs (effective number: 78.9) on 28 loci. The minimum p-value of 5.53E-05 was observed for rs10999435 located at 13 kbp 5′ of the perforin 1 (*PRF1*) gene on chromosome 10q22 (Fig. 2). Most of the genes detected in the pairwise comparisons were identified in the ordinal analysis.

### Functional evaluation of three candidate SNPs

**Figure 3:**
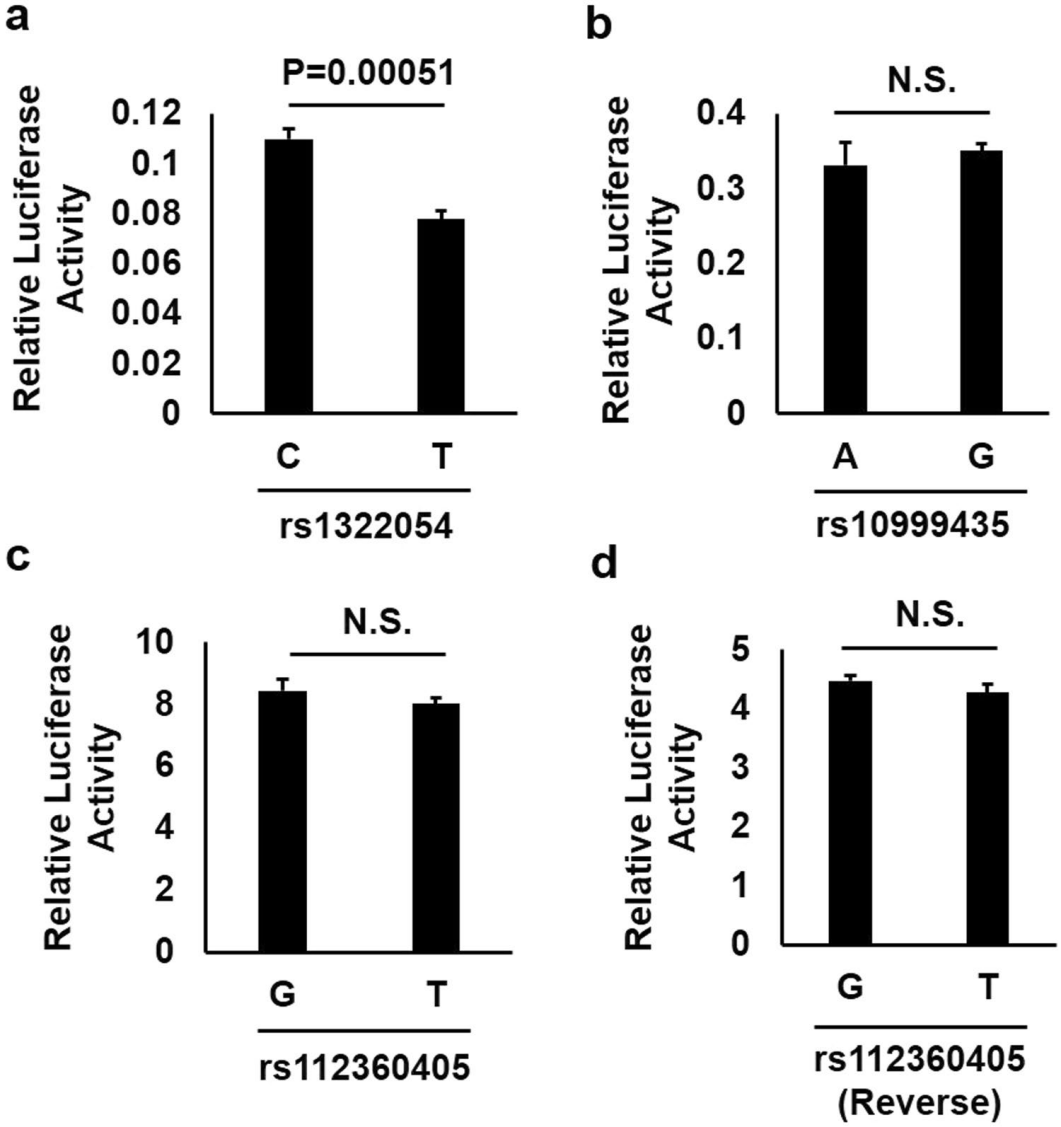
The transcriptional enhancing activity of these plasmid constructs was measured by assay of luciferase (luc) activity of the transfected human T cell line Jurkat (**a**: rs1322054; **b**: rs10999435; **c**: rs112360405 in forward direction; **d**: rs112360405 in reverse direction; respectively) 24 h after transfection. Cells transfected with the C-allele of rs1322054 showed enhanced luciferase activities compared to the T-allele of rs1322054 (*P* = 0.00051, Student’s t test). Values of relative luciferase activity are shown as mean ± SD.

In order to evaluate the functionality of candidate SNPs to regulate gene expression in immune cell, luciferase assays were performed using the human T cell line Jurkat (Fig. 3). Among the candidate SNPs above, rs1322054, rs10999435, and rs112360405 were selected based on their functional annotation in RegulomeDB database with score 2a or 2b. Among these candidate SNPs, pGL4.23 constructs of rs1322054 and rs10999435 that included DNA sequences in forward direction were prepared. Alternatively, rs112360405 was located at 5′ of *HRAS* and 5′ of *LRRC56*. Therefore, pGL4.23 constructs of rs112360405 that included DNA sequence in forward and reverse direction were prepared. As the result, luciferase activity was significantly increased by the C allele of rs1322054 compared to the T allele 18 h after transfection of the pGL4.23 vector (Fig. 3a, *P* = 0.00051). This tendency was concordant with the e-QTL data of *TNFSF8* expression in NK cells (Fig. S5). However, no difference in induced luciferase activity was observed between the major allele and the minor allele of other SNPs (Fig. 3b–d). These results indicated that rs1322054 was the primary functional variant to regulate gene expression in immune cell.

## Discussion

Here, we report the first comprehensive association study to identify SNPs on immune- or DNA repair-related genes associated with the integration patterns of HPV in cervical cancer patients. Although several genome-wide association studies (GWASs) of cervical cancer development or HPV seropositivity have been reported^19,20^, the present study is the first to identify host genetic factors that can affect the integration of HPV DNA.

The most significant association observed in the present study was for rs10999435 based on the ordinal comparison of the three integration patterns. This SNP is located upstream of the *PRF1* gene, and is predicted to generate a transcription regulatory element characterized by a DNaseI hypersensitivity cluster and transcription factor binding with a score of 2a on the RegulomeDB database. The endogenous expression level of *PRF1* in whole blood was decreased by a minor allele homozygous genotype (Fig. S6), although the difference was not statistically significant because of the low frequency of the minor allele in the European population in the eQTL database compared with the Asian population (0.07 in Europeans and 0.57 in Asians). No eQTL for *PRF1* has been registered within a 50 kpb region upstream and downstream of the *PRF1* gene in the Japanese eQTL database iMETHYL, which integrates expression data for monocytes, CD4-positive T cells, and neutrophils^16^, and no significant difference was observed in our luciferase assay using Jurkat cell. Further eQTL analysis using Asian-derived immune cells is necessary to confirm the eQTL association of this SNP with *PRF1* gene expression levels. PRF1 is one of the main cytolytic proteins of cytolytic granules, and it is a key effector molecule for T-cell- and natural killer-cell-mediated cytolysis. Upregulation of Perforin 1 mediated by CD8-positive T cells after antigen-specific stimulation contributes to the control of viral replication in various viral infections, such as HIV, cytomegalovirus, and adenovirus^21–23^. Both GZMA and PRF1 are overexpressed on cytotoxic CD8 + T-lymphocytes and NK T-cell activation. These two molecules strongly correlated each other and are known to be overexpressed when tumor shows responses to immunotherapy using anti-CTLA-4 or PD-L1 inhibitors. Using GZMA and PRF1 as intratumoral immune cytolytic denominator, Roufas *et al*.^24^ examined immune cytolytic activity of 32 solid tumors using the Genomic Data Commons (GDC) Data Portal (The Cancer Genome Atlas, TCGA program3) and the GTEx web portal (Genotype-Tissue Expression project4). Cervical cancer was shown the highest immune cytolytic index along with kidney and lung. Cytolytic activity has been previously shown to correlate with oncogenic viruses such as with HPV in cervical cancer, with EBV infection in stomach cancer, and with HBV and HCV infection in liver cancer^24^, and our results of different PRF1 genotypes in the order of group A, B, and C may suggest the possibility of different amount of tumor infiltrating lymphocytes due to different availability of HPV viral antigen.

The minimum p-value in the comparison of integration patterns A vs. B, and A vs. B + C was observed on chromosome 9q32-33, and rs1322054 was predicted to generate a transcription regulatory element with a score of 2b on the RegulomeDB database and its C allele increased enhancer/suppressor activity compared to T allele in our luciferase assay. Three known immune-related genes (*TNFSF8*, *TNFSF15*, and Tenascin C) are located in this locus, and the cis-eQTL association of rs1322054 with the *TNFSF8* gene is registered in DICE database and blood eQTL browser^14,15^. Recently published article also reported rs1322054 as one of the TNFSF8 regulatory variants with the function of TNFSF8 as general controller of inflammatory responses^25^. TNFSF8 (also known as CD153) is a cytokine that promotes cell proliferation in some lymphoma cell lines, whereas it induces cell death and suppresses cell proliferation in other lymphoma cell lines. The engagement of TNFSF8 expressed on the B cell surface plays an inhibitory role in modulating immunoglobulin class switch recombination (CSR), and CSR triggers humoral immunity against papillomavirus^26^. Interestingly, *TNFSF8* was previously shown to be highly upregulated by HPV 16 E2 expression in cells^27^. The interaction between TNFSF8-mediated immunity and HPV might affect the physical status of HPV DNA in patients with cervical cancer.

**Figure 4:**
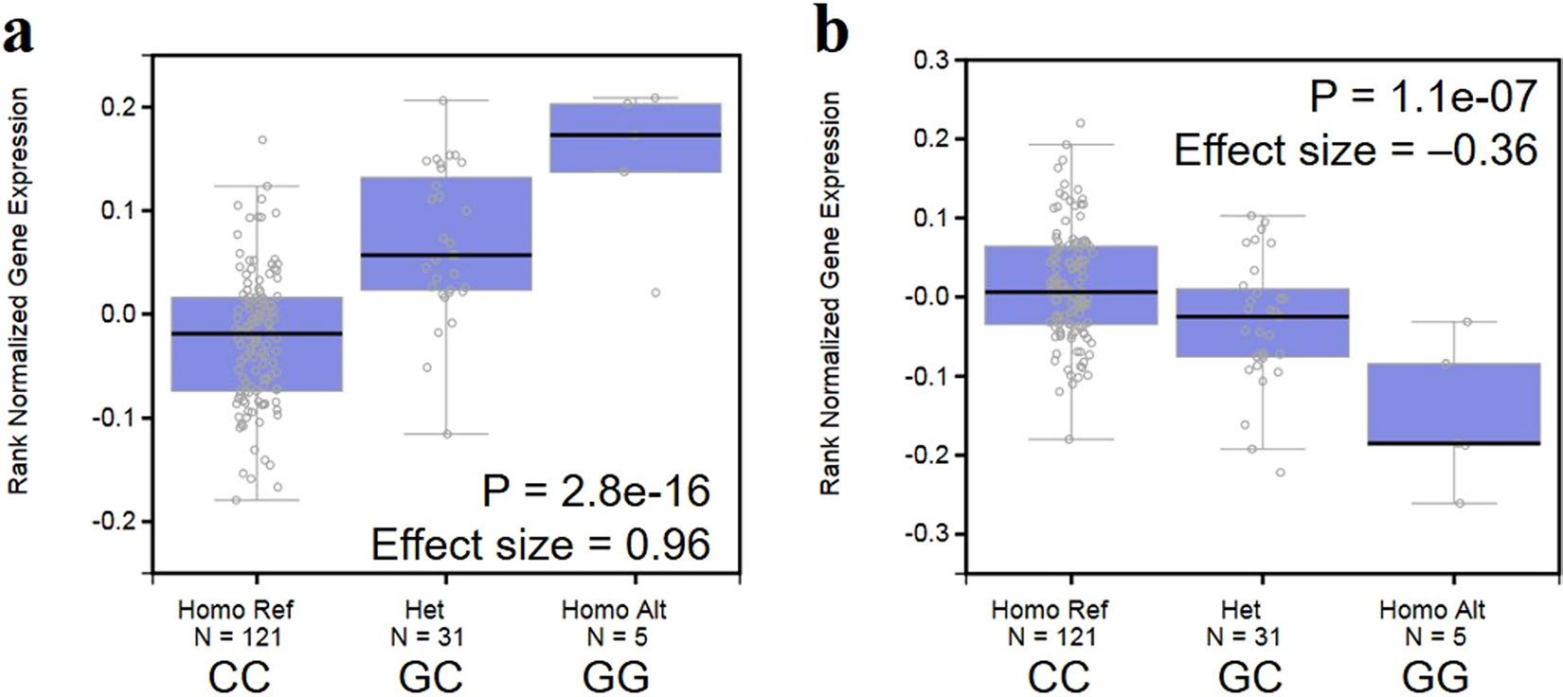
Expression quantitative trait locus (eQTL) analysis of rs10902171 with HRAS (**a**) and LRRC56 (**b**) gene expression in testis. Endogenous expression data of HRAS and LRRC56 were extracted from the GTEx portal database. Ref: reference allele, Het: heterogenous, Homo: homogenous, Alt, alternate allele.

The most significant result of the comparison between groups A vs. C, and B vs. C was observed on chromosome 11p15. Although the targeted gene based on known immune- or DNA repair-related functions was HRAS, the most significant SNP, rs10902171, induced an amino acid substitution of the LRRC56 protein with potentially damaging consequences. Although the eQTL data for cervix tissues is limited, strong eQTL associations of rs10902171 and its surrounding SNP rs112360405, which was predicted to affect a transcription regulatory element, were observed in testis against both *HRAS* and *LRRC56* (Fig. 4a,b). No significant difference was observed in our luciferase assay using Jurkat cell with evaluating both forward and reverse direction. LRRC56 is mainly expressed in testis based on the Human Protein Atlas database; however, no functional study of LRRC56 in testis and cervix has been reported. On the other hand, HRAS is a well-characterized oncogene that functions in signal transduction pathways through its intrinsic GTPase activity. Defects in *HRAS* are implicated in a variety of cancers, including bladder cancer, follicular thyroid cancer, and oral squamous cell carcinoma. HPV-positive human cervical carcinoma-derived cell lines (HeLa) stably transfected with the *HRAS* gene with its activating mutation progress through the cell cycle faster than control cells by reducing the G1 phase, indicating that HRAS could play an important role in the development of cervical cancer together with the presence of HPV^28^. Although the identified intergenic variant was independent from the reported activating mutation of the *HRAS* gene, different expression levels of *HRAS* may affect the stability of HPV DNA in the cell. HRAS is located in short arm of chromosome 11 and loss of heterozygosity (LOH) at 11p is frequently observed in cervical cancer^29^. In one report where 38 cervical cancer samples were analyzed for LOH, 11p and 11q was 16% and 22%, respectively, in its and 3 out of 5 samples which have LOH at 11p was HPV-negative^30^. It had been reported that loci on the short arm of chromosome 11 between 11p11 and 11p15 where HRAS gene is located are likely to be involved in regulation of the HPV-16 enhancer-promoter strength and this may suppress HPV-16-induced transformation by down-regulating the activity of the viral early promoter^31^. If tumors that have functional segment of chromosome 11, the activity of the viral early promoter should be down regulated, thereby suppressing HPV-induced transformation. If this segment of chromosome is not functional in cervical carcinoma, this suppression does not occur, and may show more unfavorable outcome because viral early promoter activity would not be suppressed. Our results on different distribution of HRAS genotype between HPV-negative (group C) and HPV-positive (group A and B) group may be associated with aggressive clinical outcome of patients with HPV-negative tumors. Further *in vitro* studies are required to determine the primary gene controlling the integration status of HPV.

Possibly due to the small sample size and small number of disease progression, none of the candidate SNPs showed significant association with the patients’ survival outcome (data not shown). The HPV integration pattern is a promising biomarker which was shown to be associated with patients’ survival outcome, and the goal of the current study was to examine genetic difference between patients with differential HPV integration patterns. Further larger cohort study is required to investigate SNPs directly associated with patients’ survival outcome.

In conclusion, the present comprehensive genetic association study of Korean patients with cervical cancer identified potential markers associated with the HPV integration pattern in patients with cervical cancer. Further confirmation studies are necessary.

## Supplementary information

Table S1

Table S2
